# Influence of the Milking Units on the Pulsation Curve in Dairy Sheep Milking

**DOI:** 10.3390/ani10071213

**Published:** 2020-07-17

**Authors:** Maria Caria, Giuseppe Todde, Antonio Pazzona

**Affiliations:** Department of Agricultural Science, University of Sassari, 07100 Sassari, Italy; pazzona@uniss.it

**Keywords:** liners, machine milking, pulsation chamber, working vacuum, pulsator rate, dairy sheep

## Abstract

**Simple Summary:**

In dairy farms, the mechanical milking process represents one of the most delicate activities as it may affect the quantity and quality of the milk produced and the animals’ welfare. Mechanical milking systems can be designed with different components (milking units, liners, etc.) and configured with different operative parameters (vacuum level, pulsator rate, etc.) that may significantly influence the milking performances. Thus, the aim of this study was to analyze nine milking units to evaluate the effects of the volume of the pulsation chamber, the touch point pressure of the liners and the operative parameters of the milking system, on the duration of the increasing and decreasing vacuum phases of the pulsation curve. The results obtained underlined that the characteristics of the milking units affected the pulsation curve. Specifically, the pulsation chamber volume and the pulsator rate were strictly related to the duration of the phase of milking and massage. Contrary to expectations, the touch point pressure of the milking liners was not related to the length of the increasing vacuum phase and the decreasing vacuum phase (phase “a” and “c”). This study underlined how the configuration of the mechanical milking system and the characteristics of the components installed may influence the proper length of each phase of the pulsation curve.

**Abstract:**

Mechanical milking is a critical operation in ewe dairy farming where the operative parameters and the milking routine strongly influence milk production and animal welfare. The challenge in adapting dairy animals to the farm environmental conditions may cause illness and compromise the quality of the products. From this perspective, it is important to evaluate the technological and operational aspects that can influence milk quality and animal welfare. Thus, the aim of this work was to investigate the effects on the pulsation curve of several teat cup characteristics (volume of the pulsation chamber) at determined operating parameters (vacuum level and pulsator rate) recorded from nine different milking units. Moreover, the touch point pressure of different liners was measured. Data analysis showed that the sheep milking unit characteristics affected the pulsation curve significantly. The length of both the increasing vacuum phase and the decreasing vacuum phase (phase “a” and “c”, respectively), which affect the milking and massage phases, was directly related to the pulsation chamber volume (R^2^ = 0.86) and the pulsator rate. No relationship emerged between the touch point pressure and specific characteristics of the liners such as the material, the shape, the diameter, the length, or the extension of the body. Considering the delicate role that the pulsation plays in ensuring animal welfare during milking, it is important to take into account the complete configuration and operative characteristics of the milking units. This will ensure that the complex interaction between the pulsation system and the milking units is considered when planning and assembling milking systems.

## 1. Introduction

Mechanical milking is a critical operation in dairy farming where the operating parameters and the milking routine strongly influence milking labor, milk yield and animal welfare [1]. Thus, it is essential to carefully evaluate the main technological and practical aspects of the mechanical milking process. Moreover, the differences in udder and teat morphology among dairy animals make it necessary to study and evaluate species-specific milking procedures and machine parameters [2].

Among the various components of a milking system, the milking units have the greatest influence on the efficiency of milking, efficiency being seen as the complete emptying of the udder and a stable vacuum at the teat-end [3,4]. Other characteristics of the milking units are also important and influence the pulsation curve. These include the dimensions of the long and short pulse tubes, the volume of the pulsation chamber [5,6] and liners characteristics [7,8]. In fact, the pressure variance between the pulsation chamber and the liner interior during milking, in addition to the wall thickness, material and tension of the liners, affects the level of compression applied on teat tissues that helps to reduce the congestion or edema that might arise. As reported by Penry et al. [9], increasing liner compression reduces the effects of teat-end congestion, resulting in an increased milk flow rate and an increased canal cross sectional area at high levels of teat-end vacuum and milk-phase time. As also stated by Mein et al. [10], a compressive load (12 kPa) would be adequate to relieve congestion, where any additional compression provided by the collapsed liner would provide a slight benefit. Nowadays, dairy farmers have a large variety of liners to select from in order to achieve milking comfort and speed while ensuring the welfare of the animals. The main liners’ design features include several kinds of material and shapes which influence the milking performances. Moreover, Boast et al. [11] studied the variation in rubber chemistry and dynamic mechanical properties of the milking liner barrel with age and use in dairy cows.

Establishing the correct pulsator rate plays an important role in the well-being of milking animals, contributing to the prevention of edemas or congestion in the teats, reducing the number of new infections and limiting the pain, and thus discomfort, of the animals [12,13,14,15,16].

The efficiency of pulsation in ensuring the appropriate extraction of the milk and adequate massage of the teats depends on the dynamics of the pulsation curve, or, to be more precise, the duration of the increasing + milking (a + b) and decreasing + massage (c + d) phases of the curve.

The International Organization for Standardization (ISO) 5707:2007 standard [17] prescribes that for cows and water buffaloes, phase “b” (maximum vacuum phase) shall be not less than 30% of a pulsation cycle and phase “d” (minimum vacuum phase) shall be not less than 150 ms. The latter is the threshold under which there is a considerable increase in the thickness of the teats, a factor which may result in an increased probability of new infections [18,19,20]. At present there is no information on what should be the minimum times for the various phases of milking for sheep. However, given the greater sensitivity of sheep tissue, the results for cows may also be considered to be valid for sheep [21]. Normally it is advisable to limit the duration of phases “a” (increasing vacuum phase) and “c” (decreasing vacuum phase) in order to not reduce the phases of milking and massage, without, however, reducing them excessively. Too short intermediate phases do, indeed, cause a rapid fall in the vacuum inside the liners. This creates instability in the vacuum below the milking units, which could be one of the causes of mastitis [22]. In order to avoid prolonged milking time and prevent health problems, phase “a” should generally be between 15% and 20% of the whole cycle, while phase “c” should be between 12% and 15% [23].

Awareness of the influence of the milking units on the pulsation curve is of particular importance in sheep milking parlors because these often use different kinds of components when the maintenance is accomplished. Moreover, it is also important in mixed dairy farms, which raise both sheep and goat species, where the same operative parameters and milking parlor, with only one type of milking unit, are adopted.

This study presents the results of laboratory tests on nine milking units, for the mechanical milking of dairy sheep, to evaluate the effects of the volume of the pulsation chamber, the touch point pressure of the liners and the operative parameters of the milking system on the duration of the increasing and decreasing vacuum phases of the pulsation curve.

## 2. Materials and Methods

### 2.1. Milking Units

**Figure 1:**
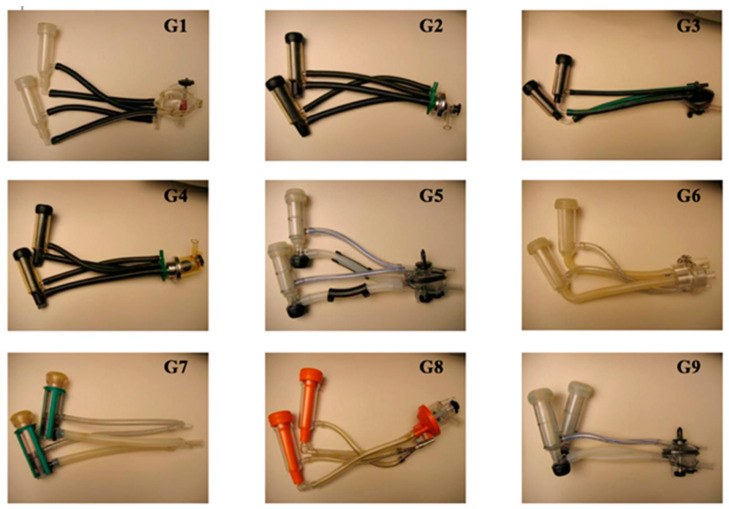
Milking units with different characteristics (**G1**–**G9**) adopted in the experimental trials.

The laboratory tests were conducted on nine milking units (Figure 1). A single type of pulsator was used (EP100 DeLaval) at a constant pulsator ratio of 50%, in order to eliminate other variable factors which were not related to the operative vacuum, the pulsator frequency and the volume of the pulsation chamber.

Among the nine milking units adopted in this study, different dimensions, shapes and materials were selected. The principal characteristics of the milking units used in the tests are shown in Table 1. A precision caliper (CN15SR, Borletti) was used to measure the dimensions of the milking units adopted in the research trials. To determine the quantity of air which entered the pulsation chamber from the pulsator, the volume of the pulsation chamber, including the short pulsation tube, was measured with the liners closed because the diameter and length of the liners was different in different models.

A pulsation tube 120 cm long and with an internal diameter of 8 mm was used in all of the tests to connect the milking units to the pulsator. The short pulsation tube where the sensors were mounted had the same dimensions as the original one.

The pulsation curves were recorded at the “Centro prove conferme metrologiche” in Maccarese (Rome), of the Associazione Italiana Allevatori (Italian Association of Breeders).

### 2.2. Liner Touch Point Pressure Tests

To evaluate the influence of the liner characteristics on the pulsation curve, the touch point pressure of the liners was measured. The touch point pressure refers to the force to be applied to the liner’s walls to cause its closure. This test was carried out by means of a universal material testing stand (Lloyd Instruments LF Plus, Bognor Regis, UK), equipped with specific ONDIO software for the analysis and processing of results. The universal material testing stand consists of a probe, a variable-speed force-driving mechanism that moves the probe, and a load cell that quantifies the material’s resistance to the force applied.

**Figure 2:**
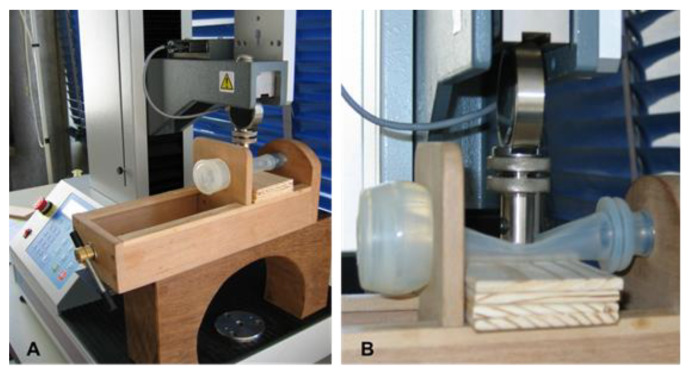
Universal material testing stand LF Plus and liner extension tool (**A**) while testing liner’s touch point pressure (**B**).

The measurement was carried out by performing a compression test (Figure 2B) in which the force parameters (measuring range between 0.5 N and 100 N) and the stand crossbar speed (500 mm/min) were previously set. To carry out the compression tests, each milking liner was removed from the shell and reassembled on an adjustable extension tool, specifically made for reproducing the original extension conditions (Figure 2A).

### 2.3. Statistical Analysis

The two-way analysis of variance with replication was applied to elaborate on the data, analyzing the operative vacuum and the pulsator rate at two levels:

- Operative vacuum, 38 kPa and 44 kPa;
- pulsator rate, 120 cycles/min and 180 cycles/min.

The test levels for each factor were chosen on the basis that these values are used in more than 90% of the 5000 milking systems used in Sardinia [25].

The data were collected after a minimum of five completed and consecutive cycles of the pulsation curve [26] for each of the milking units tested, using a combination of two vacuum levels and pulsator rates. In this way the experiment measured four hypotheses for each milking unit, for an overall total of 36 tests. The pulsator ratio in these trials was set at 50%. The data were extrapolated for the duration of the increasing and decreasing vacuum phases “a” and “c”, as these conditioned the duration of the milking and massage phases “b” and “d”. Specifically, phase “a” represents the period when the vacuum in the pulsation chamber is increasing from 4 kPa to the maximum pulsation chamber vacuum minus 4 kPa; phase “c” represents the period when the vacuum in the pulsation chamber decreases from the maximum pulsation chamber vacuum minus 4 kPa to 4 kPa [27].

A linear regression analysis was used to evaluate the influence of the volume of the pulsation chamber on the duration of phases “a” and “c”.

## 3. Results and Discussion

### 3.1. Operative Vacuum Level and Pulsation Frequency

The analysis of the data collected for each test (Table 2 and Table 3) showed that the average percentage time of phase “c” (12.9%) was shorter than phase “a” (17.7%). The combination of operative parameters that minimized the duration of the “a” and “c” phases was a vacuum of 38 kPa and a pulsator rate of 120 cycles/min, while the maximum values were registered at 44 kPa and 180 cycles/min. In tests at 120 cycles/min the average time of phases “a” and “c” were 14.3% and 10.5%, respectively. In tests at 180 cycles/min the results were 21.1% for phase “a” and 15.4% for phase “c”.

Inside these average values there was a noteworthy disparity among the various milking units. When operating at a frequency of 120 cycles/min, in about two thirds of the milking units the duration of the “a” and “c” phases was under the minimum value of 15% as recommended for dairy cows by a previous study [22]. When the pulsator rate was 180 cycles/min, the duration of phase “a” is excessive (more than 20%) in a little less than half of the milking units. The same is true for phase “c”, although to a lesser extent; the duration of phase “c” was between 12% and 15%. It seems clear that when the duration of the increasing and decreasing phases is greater than the milking and massage phases, as in the case of group G9 at 44 kPa-180 cycles/min, then the pulsation curve cannot be considered to be optimal [23]. Thus, by reducing the milking phase “b”, the total milking time will increase, while by reducing the rest phase “c”, the health status of the teat might be compromised [22].

Analysis of the variance is shown in Table 4 and Table 5. The pulsator rate has a significant effect (*p* < 0.001) on both “a” and “c” phases. On the other hand, the effects of changes in the vacuum level did not affect the two phases considered. The results highlight the lack of interaction between the vacuum and the pulsator rate. This indicates that the effects of vacuum and pulsator rate are simply additive and that when they operate in conjunction there is no additional effect.

### 3.2. Pulsation Chamber Volume and Milking Liner’s Touch Point Pressure

**Figure 3:**
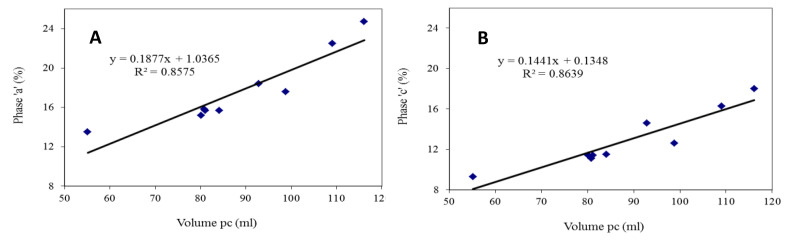
Pulsation chamber volume and length of phases “a” (**A**) and “c” (**B**) (*p* < 0.001).

The length of the “a” and “c” phases of the pulsation curve were also conditioned by the volume of air in the pulsation chamber. As can be seen from the graph in Figure 3, there was a strong correlation between the two parameters (R^2^ = 0.86 for phases “a” and “c”). For example, the length of phase “a” doubled when the volume went from 55.1 mL to 116.1 mL. Similar results were observed for phase “c”.

The force necessary to compress the liners, which was on average equal to 16.90 N, differed significantly between the models considered and was between a minimum of 12.85 N and a maximum of 20.72 N for the G6 and G5 models, respectively (Table 6). No relationship emerged between the touch point pressure and a specific characteristic of the liners such as the constituent material, the shape, the diameter, the length or extension of the body.

**Figure 4:**
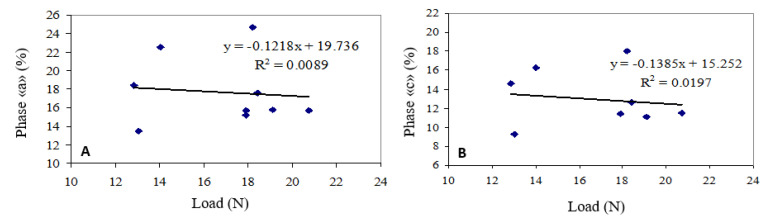
Correlation between the touch point pressure and the length of phase ”a” (**A**) and ”c” (**B**).

From the analysis of the results, the touch point pressure of the milking liners did not significantly modify the pulsation cycle, in fact the diagrams in Figure 4 show the low correlation between the two parameters (R^2^ = 0.009 for phase “a” and R^2^ = 0.020 for phase ”c”).

## 4. Conclusions

The study allowed us to define the influence of specific physical characteristics of milking units and the operative parameters of the milking system on the pulsation curve. The factors which influenced phases “a” and “c” were the pulsator rate and the volume of the pulsation chamber. Contrary to prior expectations, the touch point pressure of the milking liners did not significantly affect the pulsation, in addition, increasing the vacuum level from 38 kPa to 44 kPa did not affect the two phases considered (“a” and “c”).

Comparison of the different milking units showed that there was variation in the performance of the same pulsation system depending on the milking units used. With a pulsator rate of 120 cycles/min, in the milking units with a pulsation chamber volume of less than 85 mL, the duration of the “a” and “c” phases was too short to guarantee a suitable pulsator rate. In such conditions, there is a risk that the abrupt opening of the liners could result in the milk returning from the short tube into the teats.

Considering the delicate role that the pulsation plays in ensuring animal welfare during milking, it may be important to properly certify the complete configuration and operative characteristics of the milking units. This will ensure that the complex interaction between the pulsation system and the milking unit is taken into consideration when planning and assembling the milking systems.

## Figures and Tables

**Table 1:** Main characteristics and dimensions of the milking units adopted in the research trials.

Milking Units	Length of the Shell (mm)	Body of the Liners					Pulsation Chamber Volume (mL) ^b^
		Length (mm)	Extension (%)	Diameter (mm) ^a^	Shape	Material	
G1	103	93	10.9	190	conical	silicon	55.1
G2	104	101	2.8	200	cylindrical	rubber	80.1
G3	105	101	3.9	180	cylindrical	rubber	80.8
G4	104	101	2.8	200	cylindrical	rubber	81.1
G5	105	95	10.6	180	cylindrical	silicon	84.1
G6	109	102	7.4	18	cylindrical	silicon	92.8
G7	107	100	7.0	18	cylindrical	silicon	98.8
G8	134	124	8.5	21	conical	rubber	109.1
G9	129	114	13.2	22	cylindrical	silicon	116.1

^a^ Measured at 50 mm from the edge of the mouth [24]; ^b^ Measured with the liners closed and including the short pulsation tube.

**Table 2:** Results obtained for each milking unit tested for phase ”a” length (%) at different operative vacuum levels and pulsator rates.

Milking Units	Operative Parameters Vacuum (kPa)-Pulsator Rate (cycles/min)				Mean
	38–120	38–180	44–120	44–180	
G1	10.5	11.5	11.1	16.9	13.5
G2	11.7	17.2	12.8	19.1	15.2
G3	12.1	18.0	13.4	19.6	15.8
G4	11.9	18.1	13.1	19.7	15.7
G5	12.0	17.7	13.4	19.7	15.7
G6	16.1	20.08	15.0	21.8	18.4
G7	13.2	20.5	14.8	21.9	17.6
G8	17.9	25.8	18.7	27.6	22.5
G9	19.5	28.0	20.1	31.0	24.7
Mean ± SD	13.9 ± 3.2	20.2 ± 4.2	14.7 ± 2.9	21.9 ± 4.5	17.7 ± 3.7

**Table 3:** Results obtained for each milking unit tested for phase “c” length (%) at different operative vacuum levels and pulsator rates.

Milking Units	Operative Parameters Vacuum (kPa)-Pulsator Rate (cycles/min)				Mean
	38–120	38–180	44–120	44–180	
G1	7.3	10.8	7.7	11.4	9.3
G2	8.9	12.9	9.6	14.3	11.4
G3	8.6	12.6	9.4	13.9	11.1
G4	8.8	13.0	9.6	14.1	11.4
G5	8.8	13.0	9.8	14.4	11.5
G6	11.5	16.5	12.2	18.0	14.6
G7	9.7	14.5	10.5	15.7	12.6
G8	12.9	18.7	13.6	20.0	16.3
G9	14.2	20.8	15.1	22.0	18.0
Mean ± SD	10.1 ± 2.3	14.8 ± 3.3	10.8 ± 2.3	16.0 ± 3.4	12.9 ± 2.8

**Table 4:** Statistical Analysis of Variance results for phase “a” (%).

Source of Variation	Sum of Squares	Degrees of Freedom	Mean Square	F-Test Statistic	*p*-Value	F crit
Frequency	410.737	1	410.738	29.204	<0.001	7.499
Vacuum	14.951	1	14.951	1.063	0.310	7.499
Frequency × Vacuum	1.867	1	1.867	0.133	0.718	7.499
Within	450.055	32	14.064			
Total	877.612	35				

**Table 5:** Statistical Analysis of Variance results for phase “c” (%).

Source of Variation	Sum of Squares	Degrees of Freedom	Mean Square	F-Test Statistic	*p*-Value	F crit
Frequency	217.071	1	217.071	26.582	<0.001	7.499
Vacuum	8.801	1	8.801	1.077	0.307	7.499
Frequency × Vacuum	0.490	1	0.490	0.060	0.808	7.499
Within	261.313	32	8.166			
Total	487.675	35				

**Table 6:** Compression tests carried out on milking liners per phase “a” and “c”.

Test	Milking Liners								
	G1	G2	G3	G4	G5	G6	G7	G8	G9
Average touch point pressure (N) *	13.05 ± 0.06	17.89 ± 0.07	19.11 ± 0.06	17.89 ± 0.06	20.72 ± 0.45	12.89 ± 0.11	18.41 ± 0.19	14.02 ± 0.03	18.19 ± 0.05

* Average among four repetitions.
